# Draft Genome Sequences of Four *Pseudomonas aeruginosa* Isolates Obtained from Patients with Chronic Obstructive Pulmonary Disease

**DOI:** 10.1128/genomeA.00147-17

**Published:** 2017-06-15

**Authors:** Felipe Lira, Guillermo García-León, Antonio Oliver, José L. Martínez

**Affiliations:** a Centro Nacional de Biotecnología, CSIC, Madrid, Spain; b Servicio de Microbiología, Hospital Son Espases, Instituto de Investigación Sanitaria de Palma, Palma de Mallorca, Spain

## Abstract

Patients suffering chronic obstructive pulmonary disease are frequently infected by *Pseudomonas aeruginosa*. Nevertheless, the number of sequenced isolates causing this type of infection is low. Here, we present the draft genomes of four *P. aeruginosa* isolates obtained from patients presenting chronic obstructive pulmonary disease.

## GENOME ANNOUNCEMENT

*Pseudomonas aeruginosa* is a relevant opportunistic pathogen that is involved in several infections (1), including lung infections in cystic fibrosis (CF) patients (2) and in people presenting chronic obstructive pulmonary disease (COPD). The adaptation routes of *P. aeruginosa* causing chronic infections in CF patients are well established (3), and it has been proposed that this bacterial species plays a similar role in COPD patients (4), in which its presence is linked to acute exacerbations (5, 6). Guided by the interest in increasing the number of reference genomes for phylogenetic reconstructions and for diagnostic development, which is the aim of this study, we have sequenced and assembled the genomes of four strains of *P. aeruginosa* from COPD patients, a type of infection for which the number of sequenced *P. aeruginosa* genomes is still low.

The strains of *P. aeruginosa* sequenced in this work have been previously functionally analyzed as part of a previous study on the adaptation of *P. aeruginosa* to the lungs of COPD patients (4). They were obtained from sputum samples from COPD patients at the Hospital Son Dureta (Palma de Mallorca, Spain) during sequential exacerbation episodes. Genomic DNA of four COPD *P. aeruginosa* isolates—named COPD2a, COPD2d (presenting a hypermutable phenotype), COPD6a, and COPD6d—were extracted using the GNOME DNA isolation kit (MP Biomedicals, LLC, Illkirch, France) following the manufacturer’s instructions. Single-end sequences (1 × 75 bp) were obtained after sequencing the corresponding four libraries with an Illumina GAIIx genome analyzer. The estimated genome coverage was between 40- and 55-fold. For each strain, high-quality reads were assembled separately using the SPAdes genome assembler version 3.9.0 (http://bioinf.spbau.ru/en/spades), adjusting the “-k” parameter (-k: 21,33,55,75). Resulting contigs larger than 500 bp were submitted to gene prediction and automatic annotation using the RAST server (7, 8).

The draft genomes had an average length of 6,706,700 bp and an average G+C content of 66%. For all strains, the closest neighbors were the *P. aeruginosa* strains 19BR, 213BR, and 9BR, three isolates that present polymyxin B-adapted phenotypes (9). Our analyses highlighted the presence of antibiotic and toxic compound resistance genes in all strains. The strain with the least number of predicted resistance genes was COPD2a (59 open reading frames [ORFs]); these genes were better represented in strain COPD6d (66 ORFs). It is noteworthy that, when compared with the model PAO1 strain, all strains presented changes in the sequence of genes involved in resistance to polymyxin. All of them presented an H398R change in ParS; COPD2d, COPD6a, and COPD6d shared an L71R modification in PmrA; COPD2 and COPD6 presented a Y345H change in ParR; and COPD2a presented additional S2P, A4T, V15I, G68S, and Y345H changes in PmrB, as well as L53R and S70N modifications in ParR.

### Accession number(s).

The whole-genome shotgun projects of the four strains of *Pseudomonas aeruginosa* reported here have been deposited at DDBJ/ENA/GenBank under the accession numbers MWIE00000000 (COPD2a), MWLA00000000 (COPD2d), MWLB00000000 (COPD6a), and MWLC00000000.2 (COPD6d). The versions described in this article are versions MWIE01000000 (COPD2a), MWLA01000000 (COPD2d), MWLB01000000 (COPD6a), and MWLC02000000 (COPD6d).
